# Development of a Core Outcome Set for the research and assessment of inoperable malignant bowel obstruction

**DOI:** 10.1371/journal.pone.0289501

**Published:** 2023-08-22

**Authors:** Alison Bravington, George Obita, Elin Baddeley, Miriam J. Johnson, Fliss E. M. Murtagh, David C. Currow, Elaine G. Boland, Annmarie Nelson, Kathy Seddon, Alfred Oliver, Simon I. R. Noble, Jason W. Boland

**Affiliations:** 1 Wolfson Palliative Care Research Centre, Hull York Medical School, University of Hull, Hull, United Kingdom; 2 Dove House Hospice, Hull, England, United Kingdom; 3 Marie Curie Palliative Care Research Centre, Cardiff University, Cardiff, United Kingdom; 4 Faculty of Science, Medicine and Health, University of Wollongong, Wollongong, NSW, Australia; 5 Hull University Teaching Hospitals NHS Trust, Hull, United Kingdom; 6 National Cancer Research Institute, Consumer Liaison Group, Trans-Humber Consumer Research Panel, London, United Kingdom; Keele University & University Hospitals of North Midlands (UHNM) NHS Trust, UNITED KINGDOM

## Abstract

**Background:**

Malignant bowel obstruction is experienced by 15% of people with advanced cancer, preventing them from eating and drinking and causing pain, nausea and vomiting. Surgery is not always appropriate. Management options include tube or stent drainage of intestinal contents and symptom control using medication. Published literature describing palliative interventions uses a broad range of outcome measures, few of which are patient-relevant. This hinders evidence synthesis, and fails to consider the perspectives of people undergoing treatment.

**Aims:**

To develop a Core Outcome Set for the assessment of inoperable malignant bowel obstruction with clinician, patient and caregiver involvement, using COMET methodology (Core Outcome Measures in Effectiveness Trials).

**Methods:**

A systematic review of clinical trials and observational studies, a rapid review of the qualitative literature and in-depth patient and clinician interviews were conducted to identify a comprehensive list of outcomes. Outcomes were compared and consolidated by the study Steering Group and Patient and Public Involvement contributors, and presented to an international clinical Expert Panel for review. Outcomes from the finalised list were rated for importance in a three-round international Delphi process: results of two survey rounds were circulated to respondents, and two separate consensus meetings were conducted with clinicians and with patients and caregivers via virtual conferencing, using live polling to reach agreement on a Core Outcome Set.

**Results:**

130 unique outcomes were identified. Following the independent Expert Panel review, 82 outcomes were taken into round 1 of the Delphi survey; 24 outcomes reached criteria for critical importance across all stakeholder groups and none reached criteria for dropping. All outcomes rated critically important were taken forward for re-rating in round 2 and all other outcomes dropped. In round 2, all outcomes were voted critically important by at least one stakeholder group. Round 2 outcomes were presented again at online consensus meetings, categorised as high ranking (n = 9), middle ranking (n = 7) or low ranking (n = 8). Stakeholders reached agreement on 16 core outcomes across four key domains: Symptom control, Life impact, Treatment outcomes, and Communication and patient preferences.

**Conclusion:**

Use of this Core Outcome Set can help to address current challenges in making sense of the evidence around treatment for inoperable malignant bowel obstruction to date, and underpin a more robust future approach. Clearer communication and an honest understanding between all stakeholders will help to provide a basis for responsible decision-making in this distressing situation in clinical practice.

## Introduction

Fifteen per cent of people with advanced cancer experience obstruction of their bowel by a malignant tumour [1]. This prevents them from eating and drinking, causes pain, nausea and vomiting, and requires urgent management. Surgery is not always appropriate [2–4], and more conservative approaches may enable greater wellbeing [5,6]. Non-surgical treatments include stenting to relieve the obstruction [7,8], bowel decompression with a nasogastric tube or a gastrostomy [9,10], and medication to alleviate symptoms (anti-secretories, anti-spasmodics, anti-emetics and opioids) [11–14]. Care involves a range of disciplines, including surgery, oncology, palliative care, dietetics and specialist nursing.

Previous research has used a range of measures to assess inoperable malignant bowel obstruction, and current clinical evidence offers little guidance as to which outcomes are most appropriate. In clinical trials, the most prevalent outcomes assessed are adverse events and survival [15]. Studies assessing the key symptoms of pain, nausea and vomiting use heterogeneous measures–for example, intensity or frequency of vomiting and nausea [16,17], and intensity of pain [18,19] or time to relief of pain [20,21]. This makes it difficult to compare approaches to symptom management or aggregate data to guide treatment strategies. Given the severe impact of inoperable malignant bowel obstruction and its association with a poor prognosis, assessing how long patients survive after palliative treatment may not be the most important patient priority. There are few data to guide the choice of patient-relevant outcomes such as quality of life, and patient and caregiver perspectives remain largely unconsidered [15,22].

An established means of addressing a lack of uniformity in outcomes is to agree a Core Outcome Set (COS) of appropriate measures to be reported as a minimum in all studies in this area, to which additional measures can be added where appropriate [23]. We present the findings from a four-phase study with a published protocol [24] to develop a COS for inoperable malignant bowel obstruction for use in clinical research and routine clinical care. The scope of the study was restricted to interventions used in the palliative treatment of adults with inoperable malignant bowel obstruction, with patient-relevance as a central focus. Phases I and II combined a systematic review of clinical research [15], a separate review of qualitative research [22] and an interview study with patients and practitioners with experience of the management of inoperable malignant bowel obstruction [25] to identify a comprehensive list of outcome terms associated with the assessment of inoperable malignant bowel obstruction symptoms and treatments. Previously published results are presented in summary form in this paper, which describes the provenance of the outcome terms produced in Phases I and II and outlines the methods and results of Phases III and IV: the iterative refinement of the outcome list in order to recommend a Core Outcome Set.

## Materials and methods

**Fig 1:**
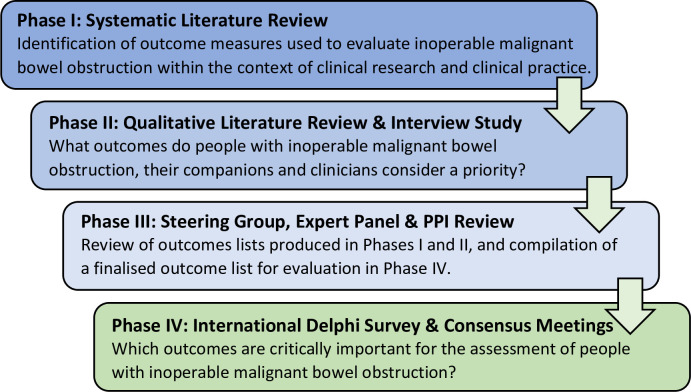
Study phases. Four-phase COS development process.

The study was prospectively registered with the COMET (Core Outcome Measures in Effectiveness Trials) initiative (http://www.comet-initiative.org/studies/details/1402) and a detailed protocol published [24]. Ethical approval for the study was obtained on 10 December 2019 from Wales Research Ethics Committee 5 Bangor (REF: 19/LO/1876). Recommendations were generated using a four-phase research design (Fig 1), and are reported in line with Core Outcome Sets-STAndards for Reporting (COS-STAR) criteria [26]. A rationale is given for the conceptualisation of outcomes into domains. Consideration is then given to how core outcomes might be measured, and implications for the application of the COS in clinical research and routine care are discussed.

### Summary of Phases I and II

Outcome terms were identified from systematic literature reviews of clinical research [15] and qualitative studies [22] and an in-depth interview study with practitioners, patients and caregivers [25].

*Phase I Systematic review of clinical research*: The review included RCTs, quasi-RCTs, single arm trials and observational studies reporting outcomes in clearly defined palliative groups of subgroups of adult patients treated for inoperable malignant bowel obstruction without concurrent chemotherapy. Data extracted included all terms used to indicate measurement of a clinical endpoint or physiological event; all items included in patient-reported outcome measures (PROMs) used in the studies were also extracted verbatim.

*Phase II Systematic review of qualitative research*: The review included all qualitative studies of adult patients undergoing palliative treatment for malignant bowel obstruction and/or their caregivers, and all qualitative studies of health care professional experiences of the management of patients with malignant bowel obstruction. Data extracted included all main themes from qualitative analysis, any terms relevant to outcome assessment, including symptom experiences/burden and quality of life, and the conclusions of each study.

*Phase II Qualitative interview study*: Recruitment for qualitative interviews took place across six UK-based NHS sites and hospices in South Wales and Hull between February and November 2020. In-depth semi-structured interviews were conducted with 7 patients and 19 health care professionals (EB, AB). One patient interview took place face-to-face in March 2020, directly prior to the COVID-19 pandemic; all subsequent interviews were conducted by telephone (24), or via virtual conferencing software (1). Written consent was taken for 24 interviews, and verbal consent was recorded for three patient interviews. Interviews were transcribed verbatim and anonymised, and data explored using thematic analysis (EB, AN, AB) [27]. Main themes exploring experiences of symptoms and treatment assessment were reviewed by the Steering Group against the outcome lists generated by the systematic reviews to identify any gaps in outcome terminology.

### Phase III: Steering Group, Expert Panel and Patient and Public Involvement (PPI) review

Steering Group and PPI Reviews: The Steering Group included three non-clinical health researchers (AB, EB, AN), seven palliative care clinicians (five with research roles) (EB, JB, DC, MJ, FEM, SN, GO), and two Patient and Public Involvement (PPI) partners (KS, AO). Team members were experienced in Patient and Public Involvement [28], which was conducted according to UK standards [29]. The group conducted two reviews of the outcome list: 1) to refine the list of outcomes produced in Phases I and II prior to the Expert Panel consultation by identifying any measures not directly related to inoperable malignant bowel obstruction or patient wellbeing; 2) to consider the Expert Panel’s comments and produce a finalised list of outcomes in plain language to take forward into Phase IV.

Expert Panel: Expert consultation took place over a period of 21 days in November 2020. Twelve authors of key papers in the systematic literature review with clinical and research experience related to the treatment and care of malignant bowel obstruction patients were invited by email to comment on terminology (clarity of definitions) and granularity (level of detail), and to identify any further potentially missing outcome measures related to inoperable malignant bowel obstruction.

### Phase IV: International Delphi survey and consensus meetings

A three-round international Delphi process was conducted to seek consensus on the relative importance of outcomes emerging from Phase III, and is reported here following Guidance on Conducting and Reporting Delphi Studies (CREDES) guidelines [30]. The survey was developed on-line on a secure Qualtrics XM™ platform at the University of York, and was piloted for face validity by two clinical members of the Steering Group, two clinicians external to the Steering Group (one oncologist and one dietitian, both with direct recent experience of malignant bowel obstruction patients), the Steering Group’s PPI lead and a PPI coordinator independent of the study team. Plain language definitions of outcome terms were produced through consultation with the team’s PPI representatives. The organisation of outcomes into categories for presentation to stakeholders was ongoing throughout the four phases of the study, and is described in detail in the results section.

Participant selection and recruitment: Steering Group members did not take part in the survey, which was administrated by non-clinical members of the research team (AB, EB). Participants were purposively recruited to ensure participation across six stakeholder groups: patients and caregivers, palliative care physicians, dieticians, oncologists, specialist nurses and surgeons. In January 2021, clinical members of the Steering Group sent invitations by email to colleagues in relevant professional networks requesting that they undertake the survey and forward the link to other relevant stakeholders. The survey link was also published on the research team’s institutional websites, and circulated on Steering Group member’s clinical and lay networks on social media. Central administrative offices of international clinical associations and charity organisations relevant to the experience or treatment of malignant bowel obstruction were contacted with a request to forward an invitation containing a survey link to members by email; this included groups related to oncology and palliative/supportive care (focusing on bowel and ovarian cancer), dietitian and specialist nursing organisations, and patient and caregiver forums. An invitation in lay language was provided for patient and caregiver organisations.

The survey introduction provided information regarding the purpose of and need for the study, the principles of COS development and what taking part would involve. Initial mandatory filter questions requested confirmation by self-report that respondents had direct experience of having or caring for/treating someone with malignant bowel obstruction, the specification of their role (professional discipline, patient or caregiver), and email contact details for round 2. The first question requested respondent’s consent for their data to be used for the survey, and only those who consented could access the subsequent survey questions. The survey administrators (AB, EB) were the only members of the research team to have access to the names and contact details of study respondents. The link for round 2 was circulated only to round 1 respondents in March 2021, and closed in April 2021.

Delphi Procedure: Round 1 introduced each of the 82 outcomes with the following question:

*Please rate how important you think it is to measure the following in people with inoperable malignant bowel obstruction: [outcome–e.g. resolution of obstruction]*

Survey respondents were asked to score each outcome for its importance on Likert scales ranging from 1 to 9 (1–3: limited importance; 4–6: important; 7–9 critically important, as proposed by the GRADE Working Group [31]). Respondents were invited to rate each outcome against two scales, following a distinction highlighted in study Phases II and III between approaches to assessing inoperable malignant bowel obstruction for research purposes and for the purpose of assessment in routine care settings. Scales were presented with the following definitions:

*RESEARCH SCALE: These outcomes will be used in research studies, so that all studies comparing treatments for inoperable IMBO can use the same measurements*.

*CLINICAL CARE SCALE: These outcomes will be used by health care professionals to measure patient outcomes in routine clinical care in hospitals, hospices and at home*.

Based on COMET guidelines [32], the prespecified definition of consensus on the critical importance of an outcome was 70% or more of panellists in the patient and caregiver group and at least two other stakeholder groups rating the outcome ≥7 and less than 15% rating it ≤3 on a 9-point Likert scale. Participants who completed round 1 (February 2021) were invited to take part in round 2 (March 2021). Results from round 1, aggregated by stakeholder group and displayed as histograms showing levels of consensus, were circulated to all respondents on the completion of each round. Individual scores were kept confidential, but made available to each respondent privately on request. Final scores for consideration at the consensus meetings were calculated using data from respondents who participated in both rounds only. The study Steering Group met regularly throughout the Delphi process to discuss the results and additional outcomes suggested by respondents in round 1, and to allow a forum for the resolution of methodological challenges.

Consensus meetings: On completion of round 2, respondents were invited to take part in a consensus meeting. Two online conferencing meetings of 1 hour were held in April 2021 on Zoom™, the first for patients and caregivers (n = 5), with the study PPI lead (KS) in attendance, and the second for clinicians (n = 11), with a co-principal investigator in attendance (SN). After consultation with PPI representatives, the decision was made to hold a separate patient and caregiver consensus meeting to facilitate more time and sensitivity in the discussion of outcomes, given the extremity of the symptoms participants had experienced or witnessed in connection with bowel obstruction, and the potential burden of on-line discussion with a larger international group of experienced clinicians. The clinician consensus meeting involved a range of disciplines with different approaches to the care of malignant bowel obstruction patients (clinical nurse specialists, dietitians, oncologists, palliative care doctors).

In each meeting, outcomes rated critically important were considered for inclusion in the COS through discussion. Delegates then voted for each outcome to be included or excluded *via* the Zoom™ anonymous live polling feature, with a consensus criterion of ≥70% agreement. Patient and caregiver consensus results were presented for consideration at the outset of the clinician consensus meeting, to enable focused and careful consideration prior to clinician voting.

## Results

**Fig 2:**
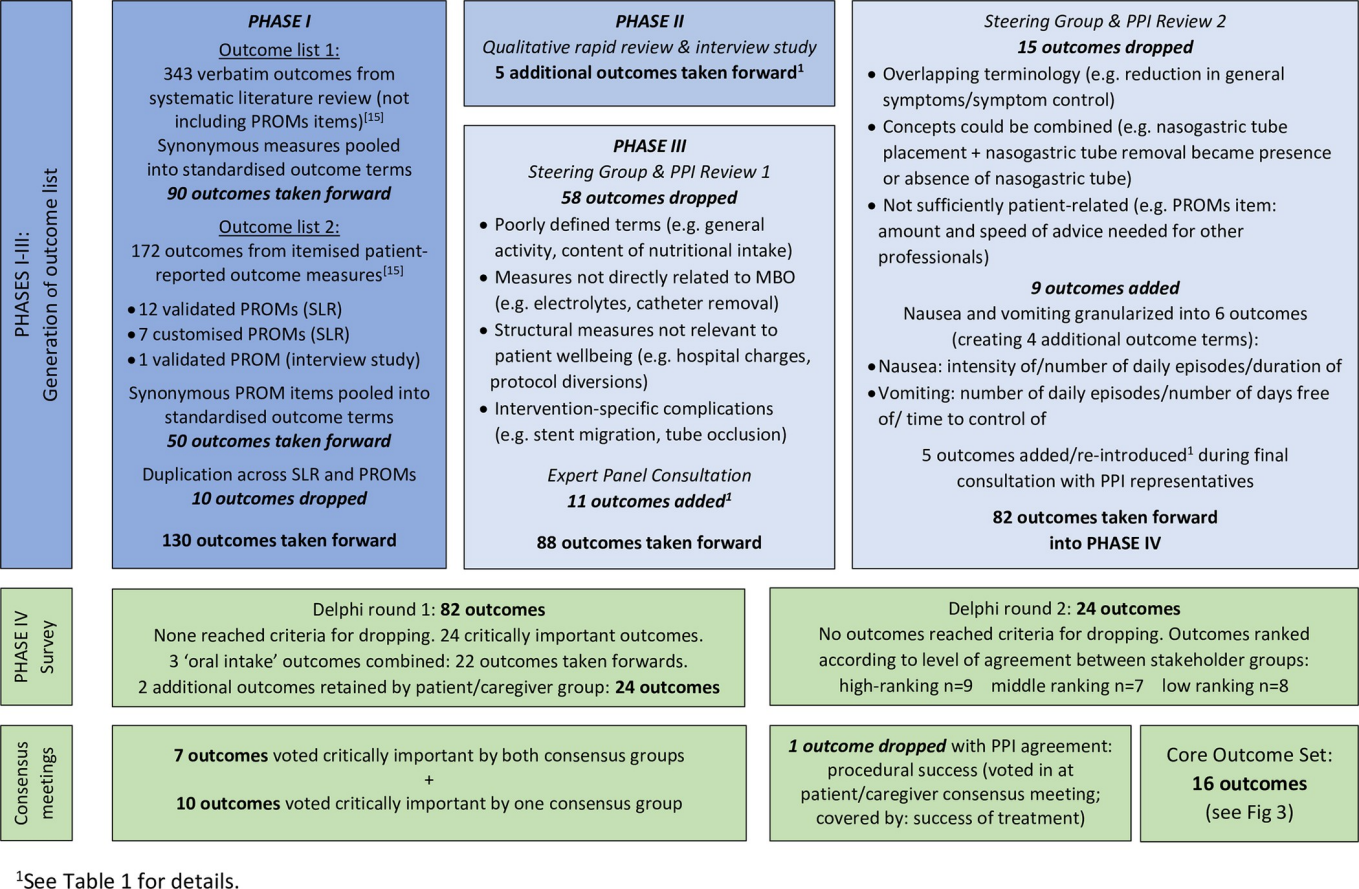
Outcomes flowchart. Outcome selection process from study inception to Core Outcome Set.

The systematic review of clinical research identified 130 unique outcome measures [15]. The systematic review of qualitative studies [22] and separate interview study [25] generated a further 5 outcomes. Fig 2 illustrates the outcome selection process from study inception to completion. A total of 135 outcomes were taken forward into Phase III.

### Phase III Steering Group & PPI Review 1

The Steering Group (including two PPI representatives) dropped 58 outcome terms on the grounds shown in Fig 2; 77 outcomes were taken forward.

### Phase III Expert Panel

Twelve specialists in malignant bowel obstruction agreed to take part in the Expert Panel to consider the outcomes proposed by the Steering Group. Five responded within the 21 days available for consultation in November 2020 (1 oncologist, 2 palliative care physicians, 1 specialist dietitian and 1 surgeon) from Australia (1), Italy (1), the UK (2) and the USA (1). The Expert Panel suggested the addition of another 11 outcomes (see Table 1), and amendments to outcome terminology.

**Table 1:** Delphi outcome list. Origin and progress of the 82 outcomes taken forward to round 1 of the Delphi survey.

No.	Origin of outcome term r = reinstated a = additional	Outcome term: Delphi Round 1	Reached Delphi Round 2	Reached Core Set: Domain no.
**PHYSICAL SYMPTOMS**				
1	SLR + PROMs	Abdominal bloating		
2	SLR + PROMs	Abdominal pain (intensity)	√	1
3	PROMs	Appetite		
4	QS (a)	Acceptable balance between side effects and reduction of symptoms (y/n)		
5	PPI review (r)	Concentration		
6	EP (a)	Discomfort in nose, throat or neck		
7	PPI review (r)	Dizziness		
8	SLR + PROMs	Drowsiness		
9	SLR + PROMs	Dry mouth		
10	EP (a)	Eating-related pain		
11	PPI review (r)	Fatigue		
	*SLR + PROMs*	*Nausea*:		
12	SG (r)	Nausea (intensity)	√	1
13	SG (r)	Nausea (number of daily episodes)		
14	SG (r)	Nausea (duration)		
15	EP (a)	Sensation of thirst		
16	PROMs	Sleep difficulties		
	*SLR + PROMs*	*Vomiting*:		
17	SG (r)	Vomiting (number of daily episodes)	√	1
18	SG (r)	Vomiting (number of days free of)		
19	SG (r)	Vomiting (time to control of)		
20	SLR	Weight loss		
21	SLR	Success of treatment as defined by clinician		
22	EP (a)	Success of treatment as defined by patient	√	3
23	SLR + PROMs	Overall symptom control	√	1
**PSYCHOLOGICAL SYMPTOMS/EFFECTS**				
24	PROMs	Ability to enjoy life	√	
25	PROMs	Anxiety/worry		
26	PROMs	Depressed mood		
27	QS (a)	Desire to eat for psychological or social comfort, despite nausea and/or vomiting		
28	PROMs	Distress	√	2
29	PROMs	Embarrassment		
30	PROMs	Mood		
31	EP (a)	Perceptions of body image		
32	PROMs	Prognostic awareness	√	
33	SLR + PROMs	Quality of life	√	2
34	PROMs	Spiritual wellbeing		
35	SLR + PROMs	Overall wellbeing	√	2
**TREATMENT-RELATED MEASURES**				
36	EP (a)	Ability to tolerate PTEG, if required		
37	SLR	Need for parenteral fluids/nutrition	√	
38	SLR	Being able to stop parenteral nutrition	√	
39	SLR	Oral intake: ability to drink fluids	√ Merged	
40	SLR	Oral intake: change from fluids only to fluids and soft food		
41	SLR	Oral intake: ability to eat solid foods		
42	SLR	Presence or absence of nasogastric tube	√	
43	SLR	Change in quantity of nasogastric aspirate		
44	EP (a)	Change in type of nasogastric aspirate		
45	SLR	Procedural success	√	
46	SLR	Resolution of obstruction	√	3
47	EP (a)	Resumption of flatus		
48	SLR + PROMs	Resumption of usual bowel function		
49	SG (a)	Palliative Surgical Outcome Score		
50	EP (a)	Avoidance of exploratory laparotomy		
51	SLR	Readmissions related to bowel obstruction	√	3
52	SLR	Surgery after recurrent symptoms		
53	SLR	Emergency surgical intervention		
54	SLR	Procedure-related complications		
55	SLR	Event-free survival	√	
**CHANGES IN PHYSICAL ABILITIES**				
56	SLR	Ability to be discharged from hospital	√	3
57	PROMs	Ability to climb stairs		
58	PROMs	Ability to make plans		
59	PROMs	Ability to self-care		
60	PROMs	Ability to undertake domestic activities		
61	PROMs	Ability to undertake recreational activities		
62	PROMs	Ability to walk		
63	PROMs	Ability to work		
64	SLR	Change in functional status		
**SOCIAL CIRCUMSTANCES**				
65	QS (a)	Family/caregiver distress in relation to patient’s inability to eat		
66	PROMs	Financial costs to patient		
67	PPI review (r)	Financial costs to family		
68	PROMs	Level of social support		
69	EP (a)	Need for support from volunteers		
70	PROMs	Support from family		
71	PROMs	Support from friends		
72	PROMs	Support from main caregiver		
**CARE-RELATED MEASURES**				
73	PROMs	Communication between health care professionals	√	4
74	PROMs	Communication between health care professionals and family caregivers		
75	PROMs	Communication between health care professionals and patients	√	4
76	PROMs	Extent of wasted time		
77	PROMs	Extent to which practical problems have been addressed		
78	EP (a)	Has a family meeting/ conference been held?		
79	PPI review (r)	Family’s understanding of treatment		
80	QS (a)	Patient’s understanding of treatment	√	4
81	QS (a)	Goals of care agreed	√	4
82	PROMs	Support from health care professionals	√	4

Key: SLR = systematic literature review of clinical research; PROMs = patient-reported outcome measures; QS = qualitative literature review and interview study; PPI = Patient and Public Involvement representatives; EP = expert panel consultation; SG = steering group review.

### Phase III Steering Group & PPI Review 2

Expert Panel comments on the outcome list were discussed in the light of issues arising in Phases I and II of the study. Fig 2 shows the criteria used by the Steering Group to refine and finalise the outcome list for presentation in the Delphi survey; 82 outcomes were taken forward. Table 1 illustrates the origin of each outcome term, and indicates outcomes that were additional to those identified in the initial systematic review and those that were initially dropped from the list and reinstated using rephrased terminology during the consultation process described above.

### Delphi survey round 1

Round 1 received 153 completed responses across six key stakeholder groups (patients and caregivers, dietitians, nursing staff, oncologists, surgeons, nursing staff, and palliative care consultants) and a further range of mixed stakeholder roles (see Table 2 for details), with 143 responses from health care practitioners and 10 responses from patients and caregivers. Given the differences in disciplinary goals of care between practitioners (for example, surgery being oriented to procedural success, palliative care being oriented to quality of life) [33,34], and given the large number of responses from health care practitioners, the decision was made to group practitioners by profession to enable respondents to consider discipline-specific differences of response in our round 1 results, and raise any interdisciplinary issues for discussion.

**Table 2:** Survey participants. Details of survey participation by stakeholder group (‘Mixed’ group: 5 researchers, 5 gastroenterologists, 1 consultant in acute medicine, 1 dentist from nutrition support team, 1 geriatrician, 1 pharmacist).

Stakeholder Group	No. of respondents, Round 1	No. of respondents, both rounds	Country															
			Australia	Brazil	Canada	France	Germany	India	Italy	Japan	Mexico	Poland	Portugal	Ireland	Spain	Switzerland	UK	USA
**TOTALS**	**153**	**88**	**5**	**1**	**3**	**1**	**1**	**3**	**3**	**7**	**1**	**2**	**1**	**1**	**3**	**1**	**49**	**6**
**By group:**																		
Patients^(p)^ & caregivers^(c)^	10 (5p, 5c)	8 (4p, 4c)															8	
Palliative Care Doctors	52	29	2		3	1	1	1	1	2		1		1	1		11	4
Dietitians	25	15															15	
Oncologists	20	14	1	1				1	2	2	1		1		1		3	1
Nurse Specialists	18	8	1													1	6	
Surgeons	14	7								1							6	
Mixed	14	7	1					1		2		1			1			1

Of the 82 outcomes presented, 8 reached the ≥70% agreement criterion for critical importance across all stakeholder groups on both scales, and a further 16 outcomes reached the agreement criterion across all stakeholder groups on at least one scale (see S1 File). In the *Care-related measures* category, two additional outcomes which did not reach the agreement criterion for critical importance across all groups reached 100% agreement within the patient and caregiver stakeholder group on at least one scale. No outcomes reached the criterion for dropping outcomes on both scales (≥70% agreement on limited importance and ≤15% agreement on critical importance).

*Post hoc* deviation from protocol: Twenty-four outcomes reaching the prespecified criteria for critical importance exceeded the GRADE working group recommendation of up to seven items for inclusion in a core outcome set [23]. Stakeholders lacked consensus over which outcomes could be dropped– 78 outcomes were rated as critically important by at least one stakeholder group in round 1; of the four outcomes that did not reach consensus with any stakeholder group in round one, only three met the ≤15% criteria for dropping items in any one stakeholder group.

The Steering Group met to discuss these issues before commencing round 2, and considered that 24 core outcomes from round 1 alone would be an unmanageable number of measures to administrate in clinical practice with palliative patients with potentially little time left to live, many of whom would have presented as an emergency. Time and capacity for the project would not allow us to conduct more than two survey rounds and one round of live voting, and the Steering Group considered the Delphi process in danger of failing to distinguish assessment measures of key importance for malignant bowel obstruction.

Other challenges discussed included 55% dropout from the survey for respondents who answered ‘yes’ to the initial filter question confirming that they had experience of malignant bowel obstruction but failed to complete round 1. Respondents who completed round 1 and left feedback felt challenged by the length of the survey and the difficulty of distinguishing between the importance of outcomes across two scales (research and clinical care)–challenges which have been reflected other studies [35]. A second round considering the remaining 58 outcomes was likely to lead to further attrition, potentially add further items to the core set, and increase the workload for the final consensus groups.

Given the remaining time available to complete the consensus process (8 weeks) the Steering Group made the decision to revise the criteria for taking outcomes forward, and to consider the survey items rated as critically important across all stakeholder groups in round 1 as distinguishing the focus for the final core set. The decision was made to ask respondents to re-rate the 24 outcomes that had reached consensus on both scales across all stakeholder groups, to reconsider their importance with a view to dropping further outcomes rather than adding additional outcomes, and to prioritise patient and caregiver representation by taking forward two further outcomes that had reached 100% consensus on at least one scale in this stakeholder group (but not across all groups) to allow their reconsideration by all respondents. The decision was made to combine the three oral intake outcome terms into a single measure, and a total of 24 outcomes were taken forward. Round 2 items were rated on a single 9-point scale. Given capacity and software constraints and the high level of agreement in round 1, we made the decision not to proactively offer individual results to all 153 respondents to round 1, but these were provided on request (one respondent requested a copy of their individual scores).

Respondents to round 1 additionally suggested 15 further outcomes for inclusion in round 2; these were reviewed by the Steering Group, but not taken forward (see Table 3).

**Table 3:** Additional outcomes reviewed (round 1). Additional outcomes suggested by round 1 survey respondents, with rationale for not taking forward to round 2.

Stakeholder group	Additional outcomes suggested (with rationale for not taking forward)
**Patients & caregivers**	*Cultural understanding (considered as part of included measure*, *communication)* *Satisfaction with follow-up (not applicable to palliative patients)* *Toiletry needs (not applicable to bowel obstruction patients)*
**Palliative Care Doctors**	*Abdominal cramps (covered by abdominal pain)* *Dyspnea (not specific to bowel obstruction)* *Review by nutritional support team (considered as part of included measure*, *oral intake)* *Time between diagnosis*, *main interventions and death (not directly applicable to palliative patients)* *Culturally-related nutritional problems (considered as part of included measures*, *communication and oral intake)*
**Dietitians**	*Bowel movements on resolution of obstruction (indicator for an included measure*, *resolution of obstruction)* *Satiety (not applicable to bowel obstruction patients)*
**Oncologists**	*Ability to resume disease-directed treatment (not applicable to palliative patients)* *Independence (not applicable to palliative patients)*
**Nurse Specialists**	*Communication of patient’s values (considered as part of included measure*, *goals of care agreed)* *Involvement in decision-making (considered as part of included measure*, *communication)*
**Surgeons**	*MDT discussion on best palliative approach (not considered a patient relevant outcome)*
**Mixed**	*-*

### Delphi survey round 2

Stakeholder group results for round 1 were provided to all respondents (individual respondents’ personal details were kept confidential), with an email invitation to round 2. Round 2 was completed by 88 of the original survey respondents. Scores were recalculated for round 1 based on the same sample and provided to all respondents (potential consensus meeting attendees), to consider how the same groups of respondents had voted across two rounds (see S1 File). All outcomes but one (‘Being able to stop parenteral nutrition’) were voted as critically important by at least one stakeholder group in round 2; none reached the predefined criteria for dropping outcomes. All outcomes were retained for the consensus meetings; all respondents were invited to take part in the meetings.

### Consensus meetings

Patient and caregiver consensus meeting: Five patients and caregiver respondents, all from the UK, attended the first consensus meeting. All 24 round 2 outcomes were presented again in three categories created *post hoc*: high ranking outcomes (5–7 stakeholder groups reached the ≥70% agreement criteria for critical importance), middle ranking outcomes (3–4 stakeholder groups reached the ≥70% agreement criteria) and low-ranking outcomes (0–2 stakeholder groups reached the ≥70% agreement criteria) (see Table 4). Consensus on core outcomes was reached through one round of live polling in the patient and caregiver meeting.

**Table 4:** Outcomes considered in consensus meeting. Outcomes presented in the consensus meetings, indicating level of stakeholder group agreement.

9 high-ranking outcomes *5 or more of the 7 stakeholder groups ranked these outcomes as critically important*	7 middle-ranking outcomes *3 or 4 of the 7 stakeholder groups ranked these outcomes as critically important*	8 low-ranking outcomes *2 or less of the 7 stakeholder groups ranked these outcomes as critically important*
Pain (7 groups)	Distress (4 groups)	Administration of parenteral fluids/nutrition (2 groups)
Overall symptom control (7)	Communication between health care professionals and patients (4)	Oral intake (2)
Quality of life (6)	Success of treatment, as defined by patient (3)	Presence or absence of nasogastric tube (2)
Intensity of nausea (5)	Procedural success (3)	Support from health care professionals (2)
Number of daily episodes of vomiting (5)	Readmissions related to bowel obstruction (3)	Ability to enjoy life (1)
Overall wellbeing (5)	Event-free survival (3)	Prognostic awareness (1)
Resolution of obstruction (5)	Ability to be discharged from hospital (3)	Communication between health care professionals (1)
Patient’s understanding of treatment (5)		Being able to stop parenteral nutrition (0)
Goals of care agreed (5)		

Clinician consensus meeting: Eleven clinicians attended the second consensus meeting (4 oncologists, 4 palliative care doctors, 2 nurse specialists and 1 dietitian) from Australia (1), Brazil (2), India (1), Italy (1), Mexico (1), Poland (1), Spain (1) and the UK (3). Results of the patient and caregiver consensus meeting were presented, and the ranked outcomes were then discussed. Consensus on core outcomes was reached through two rounds of live polling in the clinician meeting.

**Fig 3:**
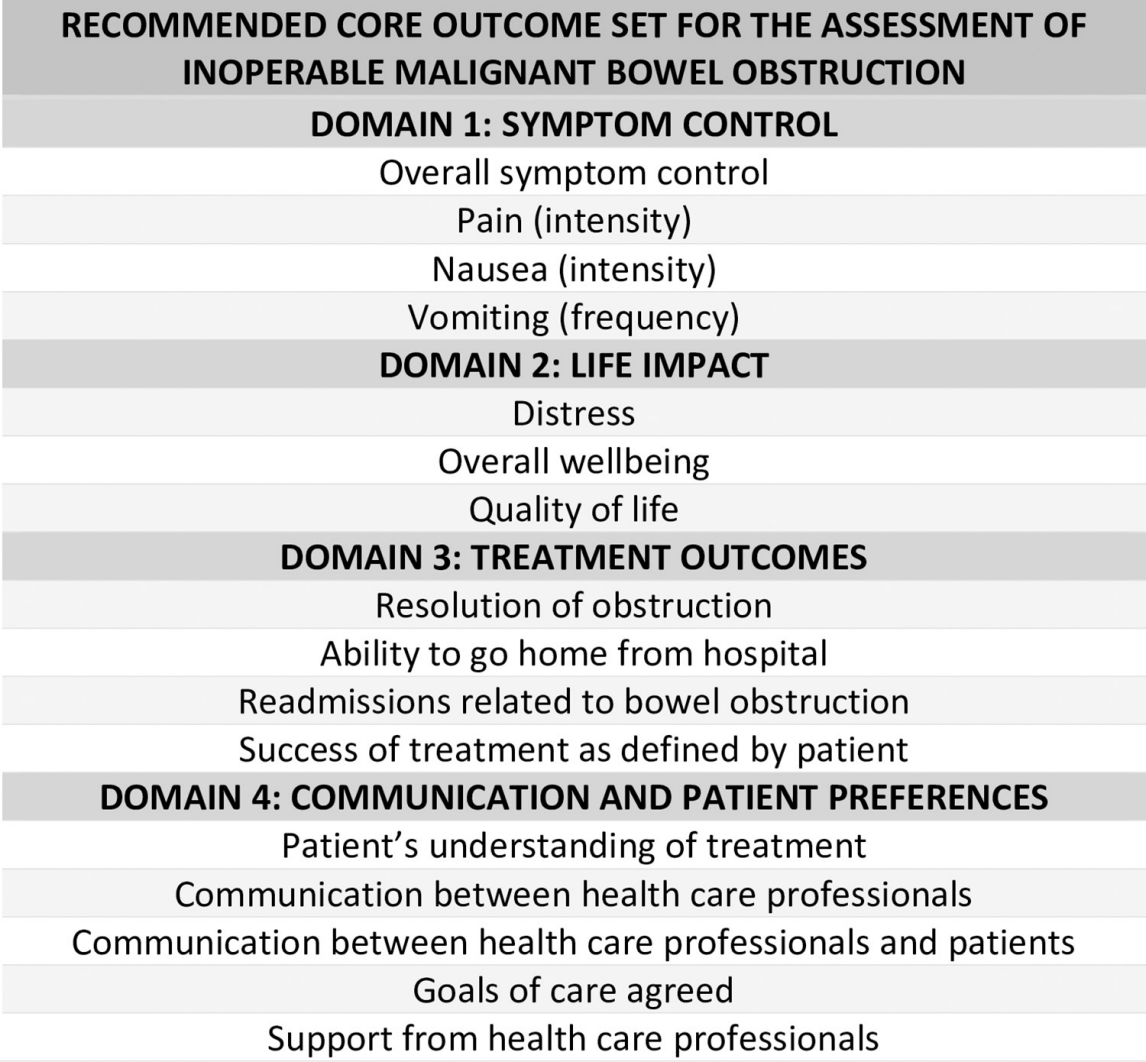
Core Outcome Set. Recommended domains and core outcomes for the assessment of inoperable malignant bowel obstruction.

Seven outcomes reached consensus for inclusion in the core set in both the clinician group and the patient and caregiver group; five additional outcomes reached consensus in the clinician group only, and five additional outcomes reached consensus in the patient and caregiver group only (see Table 5). All outcomes were taken through to the Core Set with the exception of ‘procedural success’, which was considered a surgical term that could be amalgamated with ‘success of treatment’ and applied across all intervention types. All 16 remaining outcomes are included in the COS (see Fig 3); recommendations for their use are outlined below.

**Table 5:** Results of consensus meetings. Results of live polling during the consensus meetings. Outcomes which reached consensus for inclusion in the core set in both the clinician group and the patient and caregiver group are shown in bold type.

PATIENT & CAREGIVER CONSENSUS VOTING	CLINICIAN CONSENSUS VOTING
**Outcomes reaching consensus (≥70%)**	
Pain Intensity of nausea Resolution of obstruction Communication between health care professionals Communication between health care professionals and patients Overall symptom control Quality of life Goals of care agreed Success of treatment as defined by patient Procedural success Readmissions related to bowel obstruction Support from health care professionals	**Pain** **Intensity of nausea** Number of daily episodes of vomiting **Overall symptom control** Distress **Quality of life** Ability to be discharged from hospital Patient’s understanding of treatment **Goals of care agreed** Overall wellbeing **Success of treatment as defined by patient** **Readmissions related to bowel obstruction**
**Outcomes reaching <70% consensus**	
Ability to be discharged from hospital Ability to enjoy life Administration of parenteral fluids/nutrition Event-free survival Number of daily episodes of vomiting Overall wellbeing Patient’s understanding of treatment Distress Oral intake Presence/absence of nasogastric tube Prognostic awareness Being able to stop parenteral nutrition	Ability to enjoy life Administration of parenteral fluids/nutrition Event-free survival Oral intake Presence/absence of nasogastric tube Procedural success Prognostic awareness Being able to stop parenteral nutrition Support from health care professionals

### Development of outcome domains

Consideration of taxonomies for organising the COS was ongoing throughout the four phases of the study. The COMET taxonomy [36] was considered a suitable fit for outcomes identified by Phases I and II. The 82 outcomes taken forward to round 1 of the Delphi process were recategorised for clarity in consultation with the study’s PPI representatives (KS, AO), who felt that COMET classifications may be unclear to survey respondents, and were presented in the following domains: *Physical symptoms*, *Psychological symptoms/effects*, *Treatment-related measures* (related to the measurement of procedural interventions such as tube decompression), *Changes in physical abilities* (including functional assessment), *Social circumstances* (social support and financial costs) and *Care-related measures* (relating to communication between health care professionals, patients and families). Categorisation into domains was reconsidered on the identification of the 16 outcomes voted into the Core Outcome Set; the rationale is described below.

### Recommendations for a Core Outcome Set for the assessment of inoperable malignant bowel obstruction

The 16 outcomes resulting from the consensus process were presented to the study Steering Group. Through group discussion, outcomes were categorised into four key domains. Domains 1, 2 and 3 –*Symptom control*, *Life impact* and *Treatment outcomes*–are recommended for use in clinical research. Domain 4, *Communication and patient preferences*, is intended for additional use in routine clinical care, and in studies testing interventions designed to improve communication or concordance with patient preference. Domains and outcomes are shown in Fig 3; the rationale for each domain is described below.

#### Domain 1: Symptom control

*Recommended outcomes*: Overall symptom control, pain (intensity), nausea (intensity), vomiting (frequency).

*Rationale*: Given the severity of symptoms of inoperable malignant bowel obstruction and the often acute nature of their presentation, individual measurement of the three key symptoms of pain, nausea and vomiting are recommended, in addition to an overall symptom control measure. Eating-related pain was differentiated from general abdominal pain in round 1 of the Delphi process, but only abdominal pain reached consensus for critical importance across all stakeholder groups in round 1. Only 4% of studies in our systematic literature review distinguished between types of pain (continuous or colicky); participants in our interview study highlighted colicky pain as a key issue. Pain was consistently rated by its intensity. Specification of how to measure nausea and vomiting (by frequency or severity, for example) was considered crucial, however, and three ways of measuring each of these symptoms were included in the Delphi process; intensity of nausea and frequency of vomiting were voted as critically important. In the patient and caregiver consensus meeting, we noted that type of vomiting (whether faeculent or not), may be a central concern for some caregivers–this resulted in difficulties reaching consensus on vomiting as a core outcome in the patient and caregiver consensus group.

#### Domain 2: Life impact

*Recommended outcomes*: Distress, Overall wellbeing, Quality of life.

*Rationale*: The lack of attention to life impact outcomes in the research literature, and the focus placed on these issues by patients and caregivers in the rapid review and interview study, suggest that their assessment should be of central importance for inoperable malignant bowel obstruction patients alongside the assessment of symptoms. For clinicians, the measurement of patients’ distress was a key priority. Patient-reported outcome measures distinguished between wellbeing (comfort, satisfaction with life, sense of purpose and control) and quality of life (symptoms, emotions, social support and physical/cognitive functioning), and this was supported by the Delphi findings. Patient and caregivers rated wellbeing more highly than quality of life in the final consensus meeting; clinicians rated quality of life higher than wellbeing throughout the Delphi process. The difficulties of measuring quality of life in a meaningful way in advanced cancer patients towards the end of life were acknowledged by stakeholders.

#### Domain 3: Treatment outcomes

*Recommended outcomes*: Resolution of obstruction, Ability to go home from hospital, Readmissions related to bowel obstruction, Achievement of patient-stated goals of treatment.

*Rationale*: The clinician consensus group agreed that treatment success should be tailored to the individual, but with an emphasis on clinical assessment rather than patient-stated goals. Patient and caregiver representation in steering meetings, interviews and the Delphi process emphasised a focus on the resolution of episodes of obstruction where this is possible, and on defining treatment success from the patient’s perspective. Resolution of symptoms, thereby allowing more time at home, was particularly important to patients with poor prognoses who understood that they were nearing the end of life.

#### Domain 4: Communication and patient preferences

*Recommended outcomes*: Patient’s understanding of treatment, Communication between health care professionals, Communication between health care professionals and patients, Goals of care agreed, Support from health care professionals.

*Rationale*: This domain is anticipated as particularly important for use in routine care rather than clinical research and should inform the standard for usual care. However, these would be important outcomes in studies of interventions designed to improve communication, understanding and concordance with patient preferences. The domain emerged from sustained consensus on the critical importance of communication in patient and caregiver responses throughout the Delphi process, with related PROMS items in the outcome list originating from the Support Team Assessment Schedule [37] (a precursor to the Integrated Palliative care Outcome Scale [38,39]). This was supported by our interviews with patients, which highlighted that they sensed uncertainty between health care professionals in how to best manage inoperable malignant bowel obstruction, and felt confused by conflicting information about their condition and treatment. They prioritised the determination of a clear set of patient-relevant objectives for palliative treatment with the understanding and agreement of the patient and caregivers wherever possible, based on clear and consistent information about the source of symptoms and the aims of treatment–in particular where patients experience multiple consultations across a range of clinical disciplines such as surgery, oncology, nutrition and/or palliative care. Qualitative data suggested that the role of the clinical nurse specialist is key in coordinating management.

### Recommendations for the selection of measures

Whilst recommendations for specific tools are beyond the scope of this COS development, we suggest the following considerations:

- Tools should be validated in the study population, and chosen for ease of use across settings and countries and to harmonise results to enable meta-analysis.
- For studies with symptom relief as the primary outcome, pain should be included and nausea and vomiting measured separately. A rationale should be provided for using uni- or multi-dimensional measures. Pain should be considered as a primary or co-primary measure or as part of a composite outcome, to signal its importance.
- Irrespective of primary outcome, a quality-of-life scale that can inform health economic evaluation should be used.
- Measures of wellbeing and distress are distinct from quality of life and are important to patients and caregivers.
- Usual care in clinical studies should be clearly defined, and the critical importance to patients and caregivers of communication, understanding and achievement of personal treatment goals should be recognised.

## Discussion

Despite advances in oncology, inoperable malignant bowel obstruction continues to be a distressing condition with symptoms that are challenging to manage. Precisely how distressing or challenging these symptoms are remains unclear since we lack a universal way of reporting them. This makes it difficult to accurately assess the severity, progression or resolution of obstruction in a way that is meaningful to both patients and healthcare professionals. This lack of a shared language creates a potential barrier to providing appropriate clinical and patient-focused care. In addition, the necessary research undertaken to find ways of treating malignant bowel obstruction may be rendered irrelevant if the recorded clinical endpoints are considered unrepresentative or inaccurate.

Our systematic review [15] and narrative synthesis [22] of clinical outcomes used for the assessment of malignant bowel obstruction demonstrated that individual symptoms such as vomiting, nausea, pain and quality of life are commonly reported, but methods of evaluation are inconsistent. Our Core Outcome Set offers a consistent approach to assessment, identifying four key domains of importance to patients, their caregivers, healthcare professionals and clinical researchers: *Symptom control*, *Life impact*, *Treatment outcomes* and *Communication and patient preferences*. These domains capture the whole-person experience of malignant bowel obstruction and will be of relevance to the holistic care of patients. It also offers the opportunity to identify key outcomes for different types of research. We believe that this Core Outcome Set questions measures used previously in clinical trials. For example, the evaluation of physiological parameters such as ‘change in volume of vomitus’ or ‘nasogastric drainage’ appears of little help to a patient who remains nauseated or in pain.

Pain intensity scored highly as a symptom of importance to patients, yet is rarely prioritised in clinical studies. Refocussing pain as an outcome may have considerable impact on data interpretation; Currow et al’s double blind, placebo controlled RCT did not meet a primary end point of ‘days free of vomiting’, but showed an increase in pain [17]. This suggests that the intervention did not improve malignant bowel obstruction symptoms and increased adverse events. In addition, all though the outcomes in Domain 4 may not be relevant for clinical studies designed to improve symptom control, quality of life or wellbeing, they emphasise the expectations of patients and caregivers with respect to a standard of routine care. Such standard care should be provided in the context of co-ordinated and skilled communication, reaching common ground and understanding between clinicians, patients and caregivers and acknowledgement of the individual’s stated goals of treatment, rather than grounded solely on a clinical assessment of what might be possible.

### Implications for research and clinical care

The Core Outcome Set produced by this study can be used across non-pharmacological and pharmacological interventions for inoperable malignant bowel obstruction, and will help to make a range of different palliative treatments comparable. Validated tools which can be harmonised for meta-analysis should be considered, and primary end-points justified clearly in the light of the intervention, the natural history of malignant bowel obstruction and the patient’s experience to this point. Given that many patients with this condition are very sick and have a poor prognosis, distinguishing between wellbeing and quality of life may be helpful, in addition to the avoidance of burdensome assessments comprised of a long list of measures. Quality of life measures should enable health economic evaluation. Different countries measure quality of life in different ways, and work is underway to make symptom and wellbeing measures comparable [40,41].

In clinical care, patient-stated critically important outcomes (intensity of pain, intensity of nausea and frequency of vomiting) should be prioritised over physiological measures (nasogastric tube drainage volume). Routine care measures in Domain 4 should be considered for studies testing interventions to improve communication, understanding and achieving patient goals of treatment, and for informing the standard of usual care in clinical practice and research where it aims to support honest common ground in clinical decision-making. Although prognosis was not a final outcome, a realistic understanding of likely outcomes is clearly relevant: a mixed-methods review of parenteral nutrition and venting gastrostomy in inoperable malignant bowel obstruction found that patients’ choice was strongly based on a belief that these would prolong survival–a belief fostered by the clinicians involved in their care, despite a lack of evidence to support this [42].

### Strengths and limitations

This large body of work has been conducted and reported in adherence to established and internationally accepted COMET methodology [24,26], with a significant amount of patient and public involvement at several stages along the research process. The involvement of a range of healthcare professionals not only geographically around the world, but also with respect to clinical specialities, has accounted for as wide a range of perspectives as possible. The number of patients and careers participating in the Delphi process comprised a small proportion of the number of overall contributors, and were all from the United Kingdom. This may be of relevance when considering cultural nuances–for example, with respect to the importance and ritual of eating. Dividing practitioners into smaller stakeholder groups, combined with attrition of respondents, led to smaller stakeholder groups in round 2, which may have allowed the results to be significantly influenced by the vote of a single participant. However, all outcomes from round 2 were taken through to the final round of discussion and voting at the final consensus meetings, using round 2 results for guidance. In the Delphi process, predetermined criteria for dropping outcomes was not reached–other studies using this methodology have reported similar methodological challenges, with 9-point Likert scales leading to a high number of outcomes reaching consensus [43], and protocol deviations to ensure an equal voice for patient representative stakeholders [35,44].

Attrition of patient and caregiver participation between Delphi rounds was minimal, and some responses strongly countered the opinions of the healthcare professionals, providing a hitherto unheard perspective. Our decision to hold separate consensus meetings for clinicians and patients/caregivers, rather than one consensus meeting for all, was based on advice from our Patient and Public Involvement representatives, who felt that a separate session would be less of a burden than a session dominated by clinical attendees, given the nature of malignant bowel obstruction and palliative treatment. One patient was unwell, and one caregiver had to arrange care worker cover to attend–the timing of the meeting had to be convenient for all meeting volunteers. Arranging a dedicated session allowed this flexibility, and enabled an opportunity for sensitive, inclusive and unlimited dialogue with this stakeholder group. The group were aware that their views were to be presented to clinicians prior to clinician voting.

Finally, we recognise that the natural history of malignant bowel obstruction represents a spectrum of symptoms and outcomes which change over time [45]. We have previously noted that most patients with this condition are too unwell to participate in qualitative interviews, focus groups or Expert Panel meetings [46]. The interview study included the perspectives of patients nearer death and of patients who were relatively well, with some having experienced symptomatic resolution, but the sample of patients who contributed to the study overall was small. The perspectives shared by bereaved caregivers who participated may mitigate this shortcoming.

## Conclusion

This Core Outcome Set for inoperable malignant bowel obstruction represents key outcomes important to patients, caregivers and health care professionals. Its use in clinical practice and research should help to address the current challenges in making sense of the evidence to date, and underpin a more robust approach in the future. This should enable clearer communication and an honest understanding between all stakeholders, and help to establish research that will support responsible, person-centred clinical decision-making in this distressing situation.

## Supporting information

S1 File Consensus scores for Delphi survey rounds 1 and 2. (PDF)

## References

[1] Winner M, Mooney SJ, Hershman DL, Feingold DL, Allendorf JD, Wright JD et al. Incidence and predictors of bowel obstruction in elderly patients with stage IV colon cancer: A population-based cohort study. *JAMA surgery*. 2013; 148(8):715–722. doi: 10.1001/jamasurg.2013.1 23740130 PMC4507521

[2] Merchant SJ, Brogly SB, Booth CM, Goldie C, Peng Y, Nanji S et al. Management of cancer-associated intestinal obstruction in the final year of life. *Journal of Palliative Care*. 2020; 35(2):84–92. doi: 10.1177/0825859719861935 31307272

[3] Cousins SE, Tempest E, Feuer DJ. Surgery for the resolution of symptoms in malignant bowel obstruction in advanced gynaecological and gastrointestinal cancer (Review). *Cochrane Database of Systematic Reviews*. 2016; Issue 1:CD002764. doi: 10.1002/14651858.CD002764.pub2 26727399 PMC7101053

[4] Moga M, Blidaru A, Casap S, Pascu A, Cobelschi C, Dima L. Medical management versus palliative surgery for bowel obstruction in ovarian cancer. *Gineco*.*eu Journal*. 2014; 10:117–119.

[5] Laval G, Marcelin-Benazech B, Guirimand F, Chauvenet L, Copel L, Durand A. Recommendations for bowel obstruction with peritoneal carcinomatosis. *Journal of Pain & Symptom Management*. 2014; 48:75–91. 24798105 10.1016/j.jpainsymman.2013.08.022

[6] Bethune R, Sbaih M, Brosnan C, Arulampalam T. What happens when we do not operate? Survival following conservative bowel cancer management. *Annals of the Royal College of Surgeons England*. 2016; 98:409–412. 10.1308/rcsann.2016.0146 PMC5209971 27055410

[7] Ye G-Y, Cui Z, Chen L, Zhong M. Colonic stenting vs emergent surgery for acute left-sided malignant colonic obstruction: A systematic review and meta-analysis. *World Journal of Gastroenterology*. 2012; 18(39):5608–5615. doi: 10.3748/wjg.v18.i39.5608 23112555 PMC3482649

[8] Fugazza A, Galtieri PA, Repici A. Using stents in the management of malignant bowel obstruction: the current situation and future progress. *Expert Review of Gastroenterology & Hepatology*. 2017; 11:633–41. doi: 10.1080/17474124.2017.1309283 28325090

[9] Aramaki T, Arai Y, Takeuchi Y, Sone M, Sato R, Bekku E et al. A randomized, controlled trial of the efficacy of percutaneous transesophageal gastro-tubing (PTEG) as palliative care for patients with malignant bowel obstruction: the JIVROSG0805 trial. *Supportive Care in Cancer*. 2020; 28:2563–2569. doi: 10.1007/s00520-019-05066-8 31494734

[10] Dittrich A, Schubert B, Kramer M, Lenz F, Kast K, Schuler U et al. Benefits and risks of a percutaneous endoscopic gastrostomy (PEG) for decompression in patients with malignant gastrointestinal obstruction. *Supportive Care in Cancer*. 2017; 25:2849–2856. doi: 10.1007/s00520-017-3700-1 28434096

[11] Obita GP, Boland EG, Currow D, Johnson MJ, Boland JW. Somatostatin analogues compared with placebo and other pharmacologic agents in the management of symptoms of inoperable malignant bowel obstruction: A systematic review. *Journal of Pain and Symptom Management*. 2016; 52(6):901–919. doi: 10.1016/j.jpainsymman.2016.05.032 27697568

[12] Klein C, Stiel S, Ostgathe C. Pharmacological treatment of malignant bowel obstruction in severely ill and dying patients: A systematic literature review. *Der Schmerz*. 2012; 26:587–599. 23052994 10.1007/s00482-012-1247-0

[13] Boland JW, Boland EG. ‘Malignant bowel obstruction’, in Chenry NI, Fallon MT, Kaasa S, Portenoy RK, Currow DC, Oxford Textbook of Palliative Medicine (6th edn), 2021: 904–917.

[14] Boland JW, Boland EG. Constipation and malignant bowel obstruction in palliative care. *Medicine*, 2022; 50(12):775–779.

[15] Bravington A, Obita G, Baddeley E, Johnson MJ, Murtagh FEM, Currow DC et al. The range and suitability of outcome measures used in the assessment of palliative treatment for inoperable malignant bowel obstruction: A systematic review. *Palliative Medicine*. 2022; 36(9):1336–1350. doi: 10.1177/02692163221122352 36131489 PMC10150264

[16] Laval G, Rousselot H. Toussaint-Martel S, Mayer F, Terrebonne É, François É et al. SALTO: A randomized, multicenter study assessing octreotide LAR in inoperable bowel obstruction. *Bulletin du Cancer*. 2012; 99(2):E1–E9. doi: 10.1684/bdc.2011.1535 22265994

[17] Currow DC, Quinn S, Agar M, Fazekas B, Hardy J, McCaffrey N et al. Double-blind, placebo-controlled, randomized trial of octreotide in malignant bowel obstruction. *Journal of Pain and Symptom Management*. 2015; 49(5):814–821. doi: 10.1016/j.jpainsymman.2014.09.013 25462210

[18] Tuca A, Roca R, Sala C, Porta J, Serrano G, González-Barboteo J et al. Efficacy of granisetron in the antiemetic control of nonsurgical intestinal obstruction in advanced cancer: A phase II clinical trial. *Journal of Pain and Symptom Management*. 2009; 37(2):259–270. doi: 10.1016/j.jpainsymman.2008.01.014 18789638

[19] Peng X, Wang P, Li S, Zhang G, Hu S. Randomized clinical trial comparing octreotide and scopolamine butylbromide in symptom control of patients with inoperable bowel obstruction due to advanced ovarian cancer. *World Journal of Surgical Oncology*. 2015; 13:50–55. doi: 10.1186/s12957-015-0455-3 25889313 PMC4372044

[20] Kubota H, Taguchi K, Kobayashi D, Naruyama H, Hirose M, Fukuta K et al. Clinical impact of treatment using octreotide for inoperable malignant bowel obstruction caused by advanced urological cancer. *Asian Pacific Journal of Cancer Prevention*. 2013; 14:7107–7110. 24460259 10.7314/apjcp.2013.14.12.7107

[21] Berger J, Lester P, Rodrigues L. Medical therapy of malignant bowel obstruction With octreotide, dexamethasone, and metoclopramide. *American Journal of Hospice and Palliative Medicine*. 2016; 33(4):407–410. doi: 10.1177/1049909115569047 25646530

[22] Baddeley E, Mann M, Bravington A, Johnson MJ, Currow D, Murtagh FEM et al. Symptom burden and lived experiences of patient, caregivers and healthcare professionals on the management of malignant bowel obstruction: A qualitative systematic review. *Palliative Medicine*, 2022; 36(6):895–911. 35260004 10.1177/02692163221081331 PMC9174615

[23] Williamson PR, Altman DG, Blazeby JM, Clarke M, Devane D, Gargon E, et al. Developing core outcome sets for clinical trials: issues to consider. *Trials*. 2012; 13: 132. doi: 10.1186/1745-6215-13-132 22867278 PMC3472231

[24] Baddeley E, Bravington A, Johnson M, Currow DC, Murtagh FEM, Boland E et al. Development of a core outcome set to use in the research and assessment of malignant bowel obstruction: protocol for the RAMBO study. *BMJ Open*. 2020; 10(6):e039154. doi: 10.1136/bmjopen-2020-039154 32595168 PMC7322279

[25] Baddeley E, Bravington A, Nelson A, Obita G, Johnson M, Currow D et al. Patient and clinician experiences of inoperable malignant bowel obstruction: A qualitative study (Poster Number B46). Abstracts from the 17^th^ World Congress of the European Association for Palliative Care (EAPC) 2021. *Palliative Medicine*. 2021; 35(1 Suppl), doi: 10.1177/02692163211035

[26] Kirkham JJ, Gorst S, Altman DG, Blazeby JM, Clarke M, Devane D et al. Core Outcome Set-STAndards for reporting: The COS-STAR statement. *PLOS Medicine*. 2016; 13(10):e1002148. doi: 10.1371/journal.pmed.1002148 27755541 PMC5068732

[27] Braun V and Clarke V. Using thematic analysis in psychology. *Qualitative Research in psychology*. 2006; 3(2);77–101.

[28] Seddon K, Elliot J, Johnson M, White C, Watson M, Nelson A et al. Using the United Kingdom standards for public involvement to evaluate the impact of public involvement in a multinational clinical study. *Research Involvement and Engagement*. 2021; 7:22. doi: 10.1186/s40900-021-00264-3 33931134 PMC8088001

[29] NIHR. *UK Standards for Public Involvement*. 2019. London: National Institute for Health Research.

[30] Jünger S, Payne SA, Brine J, Radbruch L, Brearley SG. Guidance on Conducting and Reporting Delphi Studies (CREDES) in palliative care: Recommendations based on a methodological systematic review. *Palliative Medicine*. 2017; 31(8):684–706. doi: 10.1177/0269216317690685 28190381

[31] Schünemann H, Brożek J, Guyatt G, Oxman A (Eds). *Handbook for grading the quality of evidence and the strength of recommendations using the GRADE approach*. 2013. Grading of Recommendations Assessment, Development and Evaluation (GRADE) working group. Available *via* www.gradeworkinggroup.org.

[32] Williamson PR, Altman DG, Bagley H, Barnes KL, Blazeby JM, Brookes ST et al. The COMET Handbook: version 1.0. *Trials*. 2017;18(3):280. doi: 10.1186/s13063-017-1978-4 28681707 PMC5499094

[33] Klement A, Marks S. The pitfalls of utilizing “goals of care” as a clinical buzz phrase: A case study and proposed solution. *Palliative Medicine Report*s. 2020; 1(1):216–220. doi: 10.1089/pmr.2020.0063 34223479 PMC8241360

[34] Scally CP, Robinson K, Blumenthaler AN, Bruera E, Badgwell BD. Identifying core principles of palliative care consultation in surgical patients and potential knowledge gaps for surgeons. *Journal of the American College of Surgeons*. 231(1): 179–185. doi: 10.1016/j.jamcollsurg.2020.03.036 32311465 PMC7714396

[35] Young B, Bagley H. Including patients in core outcome set development: issues to consider based on three workshops with around 100 international delegates. *Research Involvement and Engagement*. 2016; 2:25. doi: 10.1186/s40900-016-0039-6 29507761 PMC5831887

[36] Dodd S, Clarke M, Becker L, Mavergames C, Fish R, Williamson PR. A taxonomy has been developed for outcomes in medical research to help improve knowledge discovery. *Journal of Clinical Epidemiology*. 2018; 96:84–92. doi: 10.1016/j.jclinepi.2017.12.020 29288712 PMC5854263

[37] Higginson I. The development, validity, reliability and practicality of a new measure of palliative care: The Support Team Assessment Schedule. Doctoral thesis. London: University College London, 1992. Available at: http://discovery.ucl.ac.uk/1317889/1/296225.pdf. Accessed June 23, 2019.

[38] Hearn J, Higginson I. Development and validation of a core outcome measure for palliative care: the Palliative care Outcome Scale. Palliative Care Core Audit Project Advisory Group. *Qualitative Health Care*. 1999; 8:219e227. doi: 10.1136/qshc.8.4.219 10847883 PMC2483665

[39] Murtagh FE, Ramsenthaler C, Firth A, Groeneveld EI, Lovell N, Simon ST et al. A brief, patient- and proxy-reported outcome measure in advanced illness: validity, reliability and responsiveness of the Integrated Palliative care Outcome Scale (IPOS). *Palliative Medicine*. 2019; 33(8): 1045–1057. doi: 10.1177/0269216319854264 31185804 PMC6691591

[40] De Wolf-Linder S, Dawkins M, Wicks F, Pask S, Eagar K, Evans CJ et al. Which outcome domains are important in palliative care and when? An international consensus workshop using the nominal group technique. *Palliative Medicine*. 2019; 33(8):1058–1068. doi: 10.1177/0269216319854154 31185812 10.1177/0269216319854154 PMC6691595

[41] Collins ES, Witt J, Bausewein C, Daveson BA, Higginson IJ, Murtagh FEM. A Systematic Review of the Use of the Palliative Care Outcome Scale and the Support Team Assessment Schedule in Palliative Care. *Journal of Pain & Symptom Management*. 2015;50(6);842–853. doi: 10.1016/j.jpainsymman.2015.07.015 26335764

[42] Patterson M, Greenley S, Ma Y, Bullock A, Curry J, Smithson J, et al. Inoperable malignant bowel obstruction: palliative interventions outcomes–mixed-methods systematic review. *BMJ Supportive & Palliative Care*. 2022; 0:1–13. doi: 10.1136/bmjspcare-2021-003492 PMC10850628 38557409

[43] De Meyer D, Kottner J, Beele H, Schmitt J, Lange T, Van Hecke A et al. Delphi procedure in core outcome set development: rating scale and consensus criteria determined outcome selection. *Journal of Clinical Epidemiology*. 2019;111:23–31. doi: 10.1016/j.jclinepi.2019.03.011 30922885

[44] Remus A, Smith V, Gutke A, Mena JJS, Mørkved S, Wikmar LN et al. A core outcome set for research and clinical practice in women with pelvic girdle pain: PGP-COS. *PLoS ONE*. 16(2): e0247466. doi: 10.1371/journal.pone.0247466 33630941 PMC7906405

[45] Boland JW, Koffman J, Boland EG. Invited Editorial: What do we do with all the evidence for symptoms in palliative care? *Palliative Medicine*, 2022; 36(6):892–894. 35658645 10.1177/02692163221098005

[46] Baddeley E, Bravington A, Nelson A, Obita G, Johnson M, Currow D et al. Exploring conditions that Render Patients too Unwell to Participate: Challenges from the RAMBO Study (Poster Number Q16). Abstracts from the 17^th^ World Congress of the European Association for Palliative Care (EAPC) 2021. *Palliative Medicine*. 2021; 35(1 Suppl), doi: 10.1177/02692163211035

