# Supplementary material for: Development of a Core Outcome Set for the research and assessment of inoperable malignant bowel obstruction
Source: PLoS One. 2023 Aug 22;18(8):e0289501. doi: 10.1371/journal.pone.0289501 (PMC10443874; doi:10.1371/journal.pone.0289501)
Supplement: S1 File — (PDF) [file pone.0289501.s001.pdf]

Wolfson Palliative Care Research Centre, Hull York Medical School, University of Hull, UK

Marie Curie Palliative Care Research Centre, Cardiff University, Cardiff, UK

Research and assessment of inoperable malignant bowel obstruction: DELPHI SURVEY

PERCENTAGE CONSENSUS ON CRITICAL IMPORTANCE (7-9) ACROSS STAKEHOLDER GROUPS

153 responses to round 1

ROUND 1: 24 OUTCOMES VOTED CRITICALLY IMPORTANT

Separate scales for assessment in research and assessment in routine clinical care.

R1 OUTCOMES		PATIENT & CAREGIVERS (10)		PALLATIVE CARE DOCTORS (52)		DIETITIANS (25)		ONCOLOGISTS (20)		SPECIALIST NURSES (18)		SURGEONS (14)		MIXED (14)	
		RESEARCH	CLIN CARE	RESEARCH	CLIN CARE	RESEARCH	CLIN CARE	RESEARCH	CLIN CARE	RESEARCH	CLIN CARE	RESEARCH	CLIN CARE	RESEARCH	CLIN CARE
	**PHYSICAL SYMPTOMS**														
1	Abdominal bloating	60	90	45	68	68	92	55	60	50	89	43	36	43	57
2	Abdominal pain	60	90	83	94	96	100	90	100	94	100	79	79	86	93
3	Appetite	40	60	42	49	60	84	50	50	33	50	29	43	50	50
4	Acceptable balance between side effects and reduction of symptoms	60	60	85	91	88	88	75	90	100	100	86	86	79	93
5	Concentration	10	40	23	26	16	12	35	50	17	17	21	0	14	86
6	Discomfort in nose, throat or neck	20	20	28	32	16	20	30	40	22	50	7	29	29	36
7	Dizziness	50	50	15	21	24	32	45	55	22	33	14	7	7	29
8	Drowsiness	40	40	32	42	32	52	55	60	50	72	21	29	29	29
9	Dry mouth	60	70	45	62	24	56	55	65	56	89	14	43	29	43
10	Eating-related pain	70	70	68	72	84	92	75	90	78	94	57	64	64	71
11	Fatigue	60	70	45	51	64	60	65	65	67	83	21	29	64	57
12	Intensity of nausea	80	80	96	98	96	100	90	85	100	100	71	71	93	85
13	Number of daily episodes of nausea	50	60	87	85	80	80	75	80	100	94	57	57	79	79
14	Duration of nausea	60	60	74	70	76	84	80	85	94	94	57	57	79	86
15	Sensation of thirst	50	60	66	76	44	60	65	85	78	83	50	50	36	43
16	Sleep difficulties	60	60	30	40	36	44	50	75	44	67	21	43	43	50

R1 OUTCOMES cont.		PATIENT & CAREGIVERS (10)		PALLATIVE CARE DOCTORS (52)		DIETITIANS (25)		ONCOLOGISTS (20)		SPECIALIST NURSES (18)		SURGEONS (14)		MIXED (14)	
		RESEARCH	CLIN CARE	RESEARCH	CLIN CARE	RESEARCH	CLIN CARE	RESEARCH	CLIN CARE	RESEARCH	CLIN CARE	RESEARCH	CLIN CARE	RESEARCH	CLIN CARE
17	Number of daily episodes of vomiting	50	70	89	94	96	96	85	100	94	94	86	100	93	100
18	Number of days free of vomiting	40	40	81	81	80	80	85	80	100	100	64	64	71	71
19	Time to control of vomiting	60	70	81	76	80	72	95	100	94	100	43	43	64	79
20	Weight loss	50	60	36	25	76	72	45	45	33	44	36	29	64	71
21	Success of treatment as defined by clinician	70	70	53	55	60	68	65	55	78	72	36	43	64	64
22	Success of treatment as defined by patient	80	90	91	94	84	92	90	90	100	100	71	71	79	79
23	Overall symptom control	80	90	89	93	96	100	95	100	94	100	93	93	93	100
	**PSYCHOLOGICAL SYMPTOMS/EFFECTS**														
24	Ability to enjoy life	70	90	72	81	80	92	70	85	89	89	64	79	64	71
25	Anxiety/worry	60	90	62	79	68	76	85	85	89	94	64	57	64	71
26	Depressed mood	40	70	70	76	56	64	90	95	83	89	50	57	71	79
27	Desire to eat for psychological or social comfort, despite nausea and/or vomiting	40	50	76	87	76	84	70	80	72	78	50	71	57	71
28	Distress	60	70	76	87	76	84	75	85	83	83	57	71	71	86
29	Embarrassment	50	60	43	47	48	48	35	60	61	67	21	29	21	50
30	Mood	60	80	66	72	68	72	55	80	61	72	43	50	57	64
31	Perceptions of body image	40	60	60	64	36	40	55	75	67	67	21	14	43	36
32	Prognostic awareness	60	80	70	89	76	80	70	95	89	94	57	85	43	71
33	Quality of life	70	80	94	93	96	100	90	95	94	100	85	100	86	86
34	Spiritual wellbeing	50	50	70	72	44	48	55	80	56	72	27	86	43	57
35	Overall wellbeing	60	70	87	89	84	84	90	95	83	94	86	86	86	86
	**TREATMENT-RELATED MEASURES**														
36	Ability to tolerate PTEG, if required	80	80	72	64	60	64	60	80	89	94	57	57	57	71
37	Administration of parenteral fluids/nutrition	80	70	79	81	96	96	75	85	72	78	71	71	93	86
38	Being able to stop parenteral nutrition	80	80	79	87	92	92	95	80	83	89	71	64	71	79
39	Oral intake: ability to drink fluids	70	80	91	96	92	92	80	90	83	89	86	100	86	93
40	Oral intake: change from fluids only to fluids and soft foods	70	60	76	85	96	96	70	85	72	78	79	79	86	86
41	Oral intake: ability to eat solid foods	60	70	77	79	96	96	55	70	67	78	79	79	79	79
42	Presence or absence of nasogastric tube	70	70	83	85	92	92	80	95	61	72	64	86	79	86

R1 OUTCOMES cont.		PATIENT & CAREGIVERS (10)		PALLIATIVE CARE DOCTORS (52)		DIETITIANS (25)		ONCOLOGISTS (20)		SPECIALIST NURSES (18)		SURGEONS (14)		MIXED (14)	
		RESEARCH	CLIN CARE	RESEARCH	CLIN CARE	RESEARCH	CLIN CARE	RESEARCH	CLIN CARE	RESEARCH	CLIN CARE	RESEARCH	CLIN CARE	RESEARCH	CLIN CARE
43	Change in quantity of nasogastric aspirate	70	80	68	68	80	84	60	70	83	83	43	57	50	57
44	Change in type of nasogastric aspirate	70	70	49	59	72	80	60	70	78	83	27	36	50	64
45	Procedural success	80	80	70	77	84	92	60	85	83	94	100	93	79	86
46	Resolution of obstruction	70	80	85	93	92	88	90	100	94	94	93	100	86	86
47	Resumption of flatus	70	80	62	64	72	76	60	70	78	94	64	64	50	64
48	Resumption of usual bowel function	80	90	76	85	76	80	85	95	83	94	64	64	64	79
49	Palliative Surgical Outcome Score	80	80	66	57	88	68	70	75	89	89	79	71	71	64
50	Avoidance of exploratory laparotomy	80	80	45	53	60	56	75	85	78	78	79	79	36	36
51	Readmissions related to bowel obstruction	70	90	85	83	84	88	80	90	94	94	79	86	86	79
52	Surgery after recurrent symptoms	60	90	66	60	84	84	60	80	94	83	71	64	57	79
53	Emergency surgical intervention	70	90	64	64	92	92	70	85	94	94	86	86	71	86
54	Procedure-related complications	80	90	79	81	76	84	80	90	94	89	79	93	64	64
55	Event-free survival	80	90	79	72	80	72	85	75	94	89	79	79	71	57
	**CHANGES IN PHYSICAL ABILITIES**														
56	Ability to be discharged from hospital	80	90	79	91	84	96	75	95	89	83	79	79	64	78
57	Ability to climb stairs	40	70	28	36	36	32	35	35	28	39	14	21	36	50
58	Ability to make plans	50	60	42	60	44	52	60	60	61	72	36	43	29	50
59	Ability to self-care	60	80	55	68	48	52	75	85	39	61	21	36	64	86
60	Ability to undertake domestic activities	40	50	36	55	32	40	70	80	6	28	7	14	43	64
50 61	Ability to undertake recreational activities	70	90	36	53	40	44	50	60	17	33	21	30	36	50
62	Ability to walk	90	90	53	62	36	44	70	85	44	56	43	43	57	79
63	Ability to work	60	70	28	40	36	32	40	40	28	44	21	29	36	57
64	Change in functional status	70	90	77	83	56	56	80	85	61	72	57	64	71	79
	**SOCIAL CIRCUMSTANCES**														
65	Family/caregiver distress in relation to patient's inability to eat	50	60	72	76	64	72	55	65	72	78	21	21	79	71
66	Financial costs to patient	70	90	53	57	36	40	65	80	61	61	36	36	64	50
67	Financial costs to family	70	80	49	57	32	36	65	75	61	78	36	36	64	57
68	Level of social support	60	80	47	62	48	56	65	80	72	78	43	64	64	86
69	Need for support from volunteers	40	60	26	38	24	32	40	50	39	50	36	43	21	36
70	Support from family	60	70	62	72	36	56	65	80	72	83	43	50	43	71
71	Support from friends	40	40	38	48	24	44	45	65	50	72	29	36	29	43
72	Support from main caregiver	90	90	72	83	40	52	50	75	79	89	50	57	71	86

	R1 OUTCOMES cont.	PATIENT & CAREGIVERS (10)		PALLATIVE CARE DOCTORS (52)		DIETITIANS (25)		ONCOLOGISTS (20)		SPECIALIST NURSES (18)		SURGEONS (14)		MIXED (14)	
		RESEARCH	CLIN CARE	RESEARCH	CLIN CARE	RESEARCH	CLIN CARE	RESEARCH	CLIN CARE	RESEARCH	CLIN CARE	RESEARCH	CLIN CARE	RESEARCH	CLIN CARE
	**CARE-RELATED MEASURES**														
73	Communication between health care professionals	80	100	62	79	52	76	55	80	94	100	43	64	36	64
74	Communication between health care professionals and family caregivers	80	90	77	89	72	76	70	90	89	100	36	71	36	57
75	Communication between health care professionals and patients	90	100	83	94	64	76	70	90	95	100	57	79	43	86
76	Extent of wasted time	30	40	53	59	48	64	55	80	79	67	43	43	36	43
77	Extent to which practical problems have been addressed	70	70	62	68	64	68	60	70	79	83	35	57	43	43
78	Has a family meeting/ conference been held?	40	40	53	68	36	44	55	75	44	72	36	64	21	43
79	Family's understanding of treatment	60	50	70	87	56	76	70	95	83	100	50	79	43	57
80	Patient's understanding of treatment	90	100	89	96	64	88	80	100	89	100	71	100	57	64
81	Goals of care agreed	80	100	79	91	64	80	90	100	72	94	57	93	86	64
82	Support from health care professionals	90	100	64	76	72	80	75	90	94	89	57	71	64	86

88 responses: Stakeholders who completed both rounds

PERCENTAGE CONSENSUS FOR CRITICALLY IMPORTANT OUTCOMES: ROUND 1

Separate scales for assessment in research and assessment in routine clinical care.

	R1 OUTCOMES	PATIENT & CAREGIVERS (8)		PALLATIVE CARE DOCTORS (29)		DIETITIANS (15)		ONCOLOGISTS (14)		SPECIALIST NURSES (8)		SURGEONS (7)		MIXED (7)	
		RESEARCH	CLIN CARE	RESEARCH	CLIN CARE	RESEARCH	CLIN CARE	RESEARCH	CLIN CARE	RESEARCH	CLIN CARE	RESEARCH	CLIN CARE	RESEARCH	CLIN CARE
	**PHYSICAL SYMPTOMS**														
1	Abdominal pain	62	100	79	93	100	100	93	100	100	100	57	57	86	86
12	Intensity of nausea	75	75	97	97	93	100	93	86	100	100	71	57	86	71
17	Number of daily episodes of vomiting	38	63	93	100	100	100	86	100	88	88	86	100	86	100
22	Success of treatment as defined by patient	75	88	90	90	67	73	100	93	100	100	57	57	71	57
23	Overall symptom control	75	88	83	90	93	100	100	100	100	100	86	86	100	100
	**PSYCHOLOGICAL SYMPTOMS/EFFECTS**														
24	Ability to enjoy life	63	88	69	83	67	87	64	79	75	86	71	71	57	57
28	Distress	50	63	76	83	73	87	79	79	88	86	57	57	71	86
32	Prognostic awareness	63	88	86	97	67	80	79	93	75	86	71	86	43	71
33	Quality of life	63	75	97	90	93	100	93	93	100	100	86	100	86	86
35	Overall wellbeing	50	63	86	79	73	73	93	93	100	100	71	71	86	71
	**TREATMENT-RELATED MEASURES**														
37	Administration of parenteral fluids/nutrition	75	63	79	83	100	100	71	79	100	100	71	86	86	86
38	Being able to stop parenteral nutrition	75	75	83	86	100	100	86	93	100	100	86	86	43	57
39	Oral intake: ability to drink fluids	75	75	90	97	93	93	86	93	75	88	86	100	86	100
40	Oral intake: change from fluids only to fluids and soft foods	88	75	76	90	100	100	71	86	63	63	86	86	86	86
41	Oral intake: ability to eat solid foods	75	75	79	79	100	100	64	71	50	63	86	86	71	86
42	Presence or absence of nasogastric tube	63	63	83	83	93	100	93	100	38	63	71	86	86	86
45	Procedural success	75	75	76	79	80	93	71	86	88	88	100	100	86	86

R1 OUTCOMES cont.		PATIENT & CAREGIVERS (8)		PALLATIVE CARE DOCTORS (29)		DIETITIANS (15)		ONCOLOGISTS (14)		SPECIALIST NURSES (8)		SURGEONS (7)		MIXED (7)	
		RESEARCH	CLIN CARE	RESEARCH	CLIN CARE	RESEARCH	CLIN CARE	RESEARCH	CLIN CARE	RESEARCH	CLIN CARE	RESEARCH	CLIN CARE	RESEARCH	CLIN CARE
46	Resolution of obstruction	63	75	93	97	93	87	93	100	88	88	100	100	71	57
51	Readmissions related to bowel obstruction	63	88	90	83	80	87	86	86	88	88	86	86	86	71
55	Event-free survival	75	88	79	76	87	67	79	71	88	88	71	71	86	86
	**CHANGES IN PHYSICAL ABILITIES**														
56	Ability to be discharged from hospital	75	88	76	86	80	93	86	93	75	88	86	86	71	86
	**SOCIAL CIRCUMSTANCES**														
-	NO OUTCOMES RATED CRITICALLY IMPORTANT														
	**CARE-RELATED MEASURES**														
73	Communication between health care professionals	75	100	66	79	33	73	50	71	88	100	43	57	29	57
75	Communication between health care professionals and patients	88	100	83	90	53	73	71	86	88	100	57	71	29	86
80	Patient’s understanding of treatment	88	100	90	93	53	93	79	100	88	100	71	100	57	57
81	Goals of care agreed	75	100	76	86	60	87	93	100	75	88	57	86	86	71
82	Support from health care professionals	88	100	62	72	67	80	71	93	88	88	71	71	71	86

PERCENTAGE CONSENSUS FOR CRITICALLY IMPORTANT OUTCOMES: ROUND 2

Single scale.

R2 OUTCOMES		PATIENT & CAREGIVERS (8)	PALLATIVE CARE DOCTORS (29)	DIETITIANS (15)	ONCOLOGISTS (14)	SPECIALIST NURSES (8)	SURGEONS (7)	MIXED (7)
	**PHYSICAL SYMPTOMS**							
1	Abdominal pain	100	90	87	100	100	86	71
2	Intensity of nausea	50	97	87	86	100	43	71
3	Number of daily episodes of vomiting	50	79	73	86	100	43	86
4	Success of treatment as defined by patient	63	69	80	86	100	43	29
5	Overall symptom control	88	72	93	86	75	71	71

R2 OUTCOMES cont.		PATIENT & CAREGIVERS (8)	PALLATIVE CARE DOCTORS (29)	DIETITIANS (15)	ONCOLOGISTS (14)	SPECIALIST NURSES (8)	SURGEONS (7)	MIXED (7)
	**PSYCHOLOGICAL SYMPTOMS/EFFECTS**							
6	Ability to enjoy life	75	45	67	50	63	57	57
7	Distress	63	69	80	79	100	43	71
8	Prognostic awareness	75	48	47	57	63	57	0
9	Quality of life	63	72	93	86	100	86	86
10	Overall wellbeing	75	52	47	93	75	71	71
	**TREATMENT-RELATED MEASURES**							
11	Administration of parenteral fluids/nutrition	88	41	73	57	25	43	43
12	Being able to stop parenteral nutrition	50	55	67	43	38	57	43
13	Oral intake	75	59	67	57	50	29	71
14	Presence or absence of nasogastric tube	50	66	80	64	50	43	71
15	Procedural success	88	59	47	71	63	71	43
16	Resolution of obstruction	88	83	53	86	63	71	86
17	Readmissions related to bowel obstruction	63	55	93	86	63	71	43
18	Event-free survival	38	35	67	86	75	57	71
	**CHANGES IN PHYSICAL ABILITIES**							
19	Ability to be discharged from hospital	50	62	73	86	63	86	57
	**SOCIAL CIRCUMSTANCES**							
-	NO OUTCOMES CRITICALLY IMPORTANT							
	**CARE-RELATED MEASURES**							
20	Communication between health care professionals	63	41	27	57	88	43	43
21	Communication between health care professionals and patients	63	62	80	71	100	71	57
22	Patient's understanding of treatment	88	55	73	86	88	71	43
23	Goals of care agreed	38	72	53	93	100	71	71
24	Support from health care professionals	75	41	47	64	88	43	43

RANKING OF ROUND 2 OUTCOMES
FOR USE IN CONSENSUS MEETINGS:

No. of stakeholder groups (out of 7) who reached 70% consensus on critical importance of outcome	OUTCOME	RANK
7	Abdominal pain<br>Overall symptom control	HIGH RANKING OUTCOMES
6	Quality of life	
5	Intensity of nausea<br>Number of daily episodes of vomiting<br>Overall wellbeing<br>Resolution of obstruction<br>Patient's understanding of treatment<br>Goals of care agreed	
4	Distress<br>Communication between health care professionals and patients	MIDDLE RANKING OUTCOMES
3	Success of treatment, as defined by patient<br>Procedural success<br>Readmissions related to bowel obstruction<br>Event-free survival<br>Ability to be discharged from hospital	
2	Administration of parenteral fluids/nutrition<br>Oral intake<br>Presence or absence of nasogastric tube<br>Support from health care professionals	LOW RANKING OUTCOMES
1	Ability to enjoy life<br>Prognostic awareness<br>Communication between health care professionals	
0	Being able to stop parenteral nutrition	
